# Supplementary material for: Determinants of stillbirth among women who delivered in hospitals of North Wollo Zone, Northeast Ethiopia: A case-control study
Source: PLoS One. 2024 Apr 11;19(4):e0301602. doi: 10.1371/journal.pone.0301602 (PMC11008825; doi:10.1371/journal.pone.0301602)
Supplement: S1 Table — (PDF) [file pone.0301602.s001.pdf]

S1 Table: Data extraction checklist

Identification related information

Data collector's Name: ______________________ Signature: ____________Date___________

Supervisor's Name: _________________________ Signature: ____________________________

Questionnaire Code No: _________________ Completion status (Completely filled or Not)

S.No	Question	Response	Skip
**Part I: Socio- demographic Characteristics**			
101	Age of the mother	/_____________/ in year	
102	Educational status of mother	1. Cannot able to read and write<br>2. Can read and write<br>3. Grade 1-8<br>4. Grade 9-12<br>5. College and above	
103	Marital status of the mother	1. Single<br>2. Married<br>3. Divorced<br>1. Windowed	
104	Religion	1. Orthodox<br>2. Muslim<br>3. Catholic<br>4. Protestant<br>5. Other	
105	Occupation of mother	1. House wife<br>2. Merchant<br>3. Government employer<br>4. Daily laborer<br>5. Other, Specify______________	
106	Place of residence	1. Urban<br>2. Rural	
**Part II: Maternal factors**			
201	Gravidity	__________________	
202	Parity	__________________	
203	Preceding birth interval	____________ (In month, if applied)	
204	Antenatal care visit?	1. Yes<br>2. No	If no skip to 208
205	If yes to Q No 204, how many ANC visits do you have?	___________ in number	
206	Did mother have taken at least two doses TT vaccination?	1. Yes<br>2. No	
207	Did mother take Iron-folate supplementation during pregnancy?	1. Yes<br>2. No	
208	Gestational age at birth that recorded on the client card	_______________(in week)	

219	Prior history of abortion	1. Yes<br>2. No	
210	Prior history of stillbirth	1. Yes<br>2. No	If no skip to 212
211	Number of stillbirth	_______________	
212	HIV status	1. Positive<br>2. Negative<br>3. Unknown	
213	Do you have STI	1. Yes<br>2. No	
214	Hypertension during Pregnancy	1. Yes<br>2. No	
215	Preeclampsia during Pregnancy	1. Yes<br>2. No	
216	Do you have PROM	1. Yes<br>2. No	
217	Do you have APH	1. Yes<br>2. No	
218	Presence of anemia	1. Yes<br>2. No	
219	Presence of DM	1. Yes<br>2. No	
220	Duration of labor	_____________ (in Hour)	
221	Do you have obstructed labor	1. Yes<br>2. No	
222	Do you have uterine rapture	1. Yes<br>2. No	
**Part III: Fetal condition**			
301	Sex of the fetus?	1. Male<br>2. Female	
302	Weight of the new born (kg)	1. __________(in Kg)	
304	Congenital structure	1. Normal<br>2. Anomalies	
305	Type of pregnancy	1. Single<br>2. Twine<br>3. Triple<br>4. Other specify ___________	
**Part IV: labor and delivery related factors**			
401	Onset of labor	4. Induced<br>1. Spontaneous	
402	Use of partograph	1. Yes<br>2. No	
403	Fetal presentation	1. Cephalic<br>2. Breach<br>3. Shoulder<br>4. Face<br>5. Other specify_________________	

404	Cord accident	1. Yes<br>2. No	If no skip to 406
405	If yes for Q404, which type	1. Cord prolapse<br>2. Cord knot<br>3. Nuchal cord<br>4. Cord compress<br>5. Other specify ____________	
406	Color of amniotic fluid	1. Liquor<br>2. Offensive<br>3. Meconium stained	
407	Labor augmentation	1. Yes<br>2. No	
408	Mode of delivery	1. SVD<br>2. CS<br>3. Instrumental	

Thank you very much!
