# Supplementary material for: Determinants of stillbirth among women who delivered in hospitals of North Wollo Zone, Northeast Ethiopia: A case-control study
Source: PLoS One. 2024 Apr 11;19(4):e0301602. doi: 10.1371/journal.pone.0301602 (PMC11008825; doi:10.1371/journal.pone.0301602)
Supplement: Attachment — Submitted filename: Point-by-point Response Still birth [PONE-D-23-22773R1].docx [file pone.0301602.s002.docx]

**Manuscript ID number:** PONE-D-23-22773R1

**Title of paper:** Determinants of Stillbirth among Women Who Delivered in Hospitals of North Wollo Zone, Northeast Ethiopia: A Case-Control Study.

**Point-by-point Response for the Editor and reviewers**

First, we would like to thank you for allowing us to submit the second revised draft of the manuscript “Determinants of Stillbirth among Women Who Delivered in Hospitals of North Wollo Zone, Northeast Ethiopia: An Unmatched Case-Control Study” for publication in PLOS ONE journal. You [Abera Mersha] have requested us to provide a response letter addressing the comment forwarded by the reviewers when submitting the revised manuscript. We appreciate the time and effort that you and the reviewers dedicated to providing feedback on our manuscript and are grateful for the insightful comments on and valuable improvements to our paper. We have incorporated all the suggestions made by the reviewers. Those changes are highlighted within the manuscript. Please see below, for a point-by-point response to the reviewers’ comments and concerns.

**Academic Editor:**

1) Please include the following items when submitting your revised manuscript:
   a. A rebuttal letter that responds to each point raised by the academic editor and reviewer(s).
   b. A marked-up copy of your manuscript that highlights changes made to the original version.
   c. An unmarked version of your revised paper without tracked changes.

**Author response:** Thank you. We included all the requested documents while submitting the revised manuscript.

**Point-by-point Response for Reviewer-2 Evaluation**

First of all, we would like to express our deepest gratefulness and thanks for providing us with such constructive comments and suggestions. We have accepted your comments and acknowledged your devoted efforts to modify the manuscript.

1) The findings also provide strength to the argument that if the identified (and amendable) interventions are implemented by the local health authorities, it might help bring down the total number of preventable stillbirths. So, the study does have merits in the local context (as well as comparable socioeconomic and geographic contexts outside Ethiopia) from an implementation point of view.

**Author response:** Thank you for your suggestions; it gives good insight for local authorities to implement in their strategic plan to minimize preventable still birth.

2) Minor corrections (I do not need to review these changes again):
   a. Fix typos in line numbers 40 (Prolonged should be prolonged), 193, 240, 280.
   b. Line 104: 28 weeks and not 28th weeks, to be consistent with the definition of cases.
   c. Line 111: Gave birth to babies, instead of delivered babies?
   d. Add a note defining gravidity categories under table 1.
   e. Line 200 reads awkward (male babies vs girls- use consistent terminology).

**Author response:** Thank you for your constructive suggestions and comments; we addressed all typo corrections in the whole section of the manuscript accordingly.

**We thank you!**
